# Comprehensive immunohistochemical analysis of N6-methyladenosine (m6A) writers, erasers, and readers in endometrial cancer

**DOI:** 10.1007/s00432-022-04083-1

**Published:** 2022-06-22

**Authors:** Damian J. Ralser, Mateja Condic, Niklas Klümper, Jörg Ellinger, Christian Staerk, Eva K. Egger, Glen Kristiansen, Alexander Mustea, Thore Thiesler

**Affiliations:** 1 Department of Gynecology and Gynecological Oncology, University Hospital Bonn, Bonn, Germany; 2 Department of Urology and Pediatric Urology, University Hospital Bonn, Bonn, Germany; 3 Department of Medical Biometry, Informatics and Epidemiology, University Hospital Bonn, Bonn, Germany; 4 Institute of Pathology, University Hospital Bonn, Bonn, Germany

**Keywords:** Endometrial cancer, m6A, Biomarker, RNA modification

## Abstract

**Purpose:**

N6-methyladenosine (m6A) is the most frequent type of messenger RNA (mRNA) modification and is implicated in diverse physiological processes. The procedure of m6A RNA modification is regulated by a dynamic interaction of writers (METTL3, METTL4, METTL14, WTAP, KIAA1429), erasers (FTO, ALKBH5), and readers (HNRNPA2B1, HNRNPC, YTHDC1, YTHDC1, YTHDF1-3). In the oncological context, alterations in m6A were identified to be critically involved in tumorigenesis, proliferation, angiogenesis, and drug resistance across diverse cancer entities including endometrial cancer (EC).

**Methods:**

In this study, we comprehensively examined the protein expression of m6A writers, readers and erasers by immunohistochemical staining in a cohort of *N* = 65 EC patients. Protein expression data were analyzed with regard to clinical outcomes.

**Results:**

We identified enhanced protein expression levels of METTL3, METTL14, FTO, HNRNPA2B1, and HNRNPC, respectively to be of prognostic value and linked to a shortened overall survival in EC.

**Conclusion:**

Overall, our study points toward dysregulated m6A modification in EC and its possibility to serve as a promising prognostic biomarker.

## Introduction

Endometrial cancer (EC) represents the most common cancer of the female genital tract worldwide and accounts for more than 66,500 newly diagnosed cases and about 12,900 cancer-related deaths in the United States per year (Siegel et al. 2021). According to Bokhman’s dualistic scheme, EC is historically classified into two types: endometrioid, estrogen-dependent (type I) EC and non-endometrioid, non-estrogen-dependent (type II) EC. Usually, type I EC displays a better prognosis than type II EC, necessitating a more extensive surgical approach and adjuvant systemic and/or radiotherapy (Bokhman 1983; Murali et al. 2014; Suarez et al. 2017). However, this traditional dualistic scheme has limitations to reflect EC heterogeneity regarding epidemiology, clinical course, histopathology and molecular tumor biology (Brinton et al. 2013; Gaber et al. 2016). Recently, The Cancer Genome Atlas (TCGA) Network has deduced a new molecular classification based on comprehensive genomic, transcriptomic and proteomic characterization of *N* = 373 EC: (i) Polymerase Epsilon ultra-mutated (POLE), (ii) microsatellite instability hyper-mutated (MSI-H), (iii) copy-number low (CN low), and (iv) copy-number high (CN high) (Kandoth et al. 2013). This new molecular classification provides a precise tumor biology driven EC subgrouping with distinct clinical courses and prognosis, thereby enabling stratification of the necessary surgical approach and adjuvant therapy. However, despite recent achievements in both, tumor biological understanding and new therapeutic approaches, the overall prognosis of EC has not improved since the 1970s (Siegel et al. 2021). This emphasizes the urgent need for a deeper understanding of tumorigenesis and identification of prognostic and predictive biomarkers to tailor an individualized therapy to each patient.

In this context, there is increasing evidence for posttranscriptional RNA modification to be critically involved in tumorigenesis, progression, and genesis of therapy resistance—thereby representing an interesting candidate for both, therapeutical and diagnostic applications (Strick et al. 2020; Gundert et al. 2021; Hagen et al. 2021). N6-methyladenosine (m6A) is the most abundant messenger RNA (mRNA) modification. Three different enzyme groups are involved in m6A modification: (i) Methylases (‘writers’; METTL3, METTL4, METTL14, WTAP, KIAA1429) catalyze the transfer of S-adenosyl methionine groups to RNA adenine bases. (ii) Demethylases (‘erasers’; FTO, ALKBH5) are involved in reversing the methylation process. (iii) ‘Readers’ (HNRNPA2B1, HNRNPC, YTHDC1, YTHDF1-3) recognize m6A RNA modifications and activate downstream regulatory pathways (Helm and Motorin 2017). Research has shown altered m6A protein expression to be involved in EC (Pang et al. 2021). The majority of data are available for the fat mass and obesity-associated protein (FTO). Increased FTO expression was demonstrated to be associated with shorter survival. Of note, FTO expression is closely related to weight gain and obesity, both representing known risk factors for EC (Smemo et al. 2014; Katz 2019).

However, sparse is known about the remaining m6A writers, erasers, and readers and their role in EC. In this study, we aimed to comprehensively elucidate the protein expression levels of m6A writers, readers and erasers by immunohistochemistry in a cohort comprising *N* = 65 EC patients with regard to overall survival (OS).

## Methods

### Patients and specimens

The retrospective study cohort included *N* = 65 patients with EC diagnosed at the University Hospital Bonn between 2003 and 2016, with available follow-up. Tissue was obtained from surgical specimen or endometrial sampling using a pipelle. The collection of tissue was performed within the framework of the Biobank initiative of the University Hospital Bonn. All patients provided written informed consent prior to collection of biomaterials.

Clinicopathological parameters are summarized in Table 1. Baseline characteristics were obtained from clinical routine documentation. Follow-up data were updated in April 2022. Histopathological diagnosis was established based on the World Health Organization (WHO) criteria. The 7th TNM classification of the Union for International Cancer Control (UICC) was referred to determine the tumor stage.

**Table 1:** Clinicopathological characteristics of the study cohort

Endometrial cancer cohort *N* = 65	
Age (years)	
Mean	64.9 ± 11.2
Min–max	41.8–89.2
Histology	
Endometrioid	60 (92.3%)
Serous-papillary	3 (4.6%)
Undifferentiated	2 (3.1%)
Follow-up (months)	
Mean	70.7 ± 46.1
Min–max	0–177
FIGO classification	
IA	23 (35.3%)
IB	18 (27.7%)
II	12 (18.5%)
IIIA	6 (9.2%)
IIIB	5 (7.7%)
Unknown	1 (1.5%)
Lymph node involvement	
Yes	10 (15.4%)
No	23 (35.4%)
Unknown	32 (49.2%)
Grading	
G1	8 (12.3%)
G2	42 (64.6%)
G3	15 (23.1%)

The study was approved by the Ethics Committee of the University of Bonn (vote: 208/21).

### Tissue microarray (TMA) construction

The TMA was generated from formalin-fixed paraffin embedded EC tissue (FFPE) specimens. To identify representative tumor areas. Sections were stained with hematoxylin and eosin (HE). Afterward, a 1 mm core biopsy (0.785mm^2^) was taken from the selected cancer nests for each case and arranged in TMA blocks.

### Immunohistochemistry

Immunostaining of METTL3, METTL4, METTL14, WTAP, KIAA1429, FTO, ALKBH5, HNRNPA2B1, HNRNPC, YTHDC1, YTHDF1,YTHDF2, and YTHDF3 was performed on TMAs using an automated staining system (BenchMark ULTRA; Ventana Medical Systems) which performed deparaffinization, pretreatment with cell conditioning buffer (CC1 buffer, pH8), and incubation with primary antibodies (FTO (1:50; Atlas Antibodies #HPA041086), ALKBH5 (1:200; Novus #NBP1-82,188), METTL3 (1:1000; Biorbyt #orb374082), METTL4 (1:40; Atlas Antibodies #HPA040061), METTL14 (1:100; Atlas Antibodies #HPA038002), WTAP (1:100; Atlas Antibodies #HPA010550), KIAA1429 (1:25; Atlas Antibodies #HPA031530), HNRNPC (1:25; Atlas Antibodies #HPA051075), HNRNPA2B1 (1:100; Atlas Antibodies #HPA001666), YTHDC1 (1:25; Atlas Antibodies #HPA036462), YTHDF1 (1:10; Biorbyt #orb179018), YTHDF2 (1:200; Biorbyt #orb39199), YTHDF3 (1:200; Biorbyt #orb374095) at 4 °C overnight. Signal detection was performed with the UltraView DAB IHC Detection Kit (Ventana).

Immunohistochemical stainings were analyzed using Olympus BX51 microscope and the Panoramic Viewer 3DHistech. Staining intensities were evaluated by two different investigators. In detail, a four-tier scoring system was applied to categorize staining intensities (0: no staining, 1: low staining, 2: moderate staining, 3: high staining). For statistical analysis, staining intensities were divided into two groups (low and high). In the presence of staining signals from different cell compartments, the predominant subcellular localization was considered for statistical analysis. Classification of the groups low vs high depending on the staining intensity as well as the subcellular localization that was considered for statistical analysis are provided for each antibody in Table 2.

**Table 2:** Summary of analyzed proteins and their associations with overall survival

Protein	Localisation	*N* (low/high)	Intensity (low/high)	HR (95% CI)	*p* value
Writer					
METTL3	Nuclear	27/37	0–1 + /2–3 +	2.26 (0.99–5.12)	**0.045**
METTL4	Cytoplasmatic	48/15	0–2 + /3 +	0.98 (0.62–5.45)	0.970
METTL14	Nuclear	25/37	0–1 + /2–3 +	2.30 (0.96–5.51)	**0.050**
WTAP	Nuclear	16/41	0–1 + /2–3 +	1.84 (0.62–5.45)	0.262
KIAA1429	Nuclear	51/11	0–1 + /2–3 +	2.15 (0.90–5.18)	0.078
Eraser					
FTO	Nuclear	25/35	0–1 + /2–3 +	2.41 (1.01–5.76)	**0.041**
ALKBH5	**Nuclear**/cytoplasmatic	42/20	0–1 + /2–3 +	1.77 (0.81–3.86)	0.145
Reader					
HNRNPA2B1	Nuclear	14/50	0–1 + /2–3 +	4.2 (0.99–17.75)	**0.033**
HNRNPC	Nuclear	33/28	0–1 + /2–3 +	2.82 (1.26–6.31)	**0.008**
YTHDC1	**Cytoplastmatic**/membraneous/nuclear	19/42	0–1 + /3–3 +	1.44 (0.59–2.28)	0.419
YTHDF1	Cytoplasmatic	30/33	0–2 + /3 +	1.07 (0.50–2.28)	0.858
YTHDF2	Cytoplasmatic	43/21	0–2 + /3 +	1.17 (0.53–2.55)	0.701
YTHDF3	Cytoplasmatic	29/32	0–1 + /2–3 +	1.87 (0.85–4.13)	0.115

In presence of staining signals from multiple cell compartments, m6A protein localization that was considered for statistical analysis is highlighted in bold. Estimated hazard ratios (HR) with 95% confidence intervals based on univariate Cox regression analyses; *p* values for the group comparison (low vs. high expression) based on log-rank tests

### Statistical analysis

Kaplan–Meier survival analyses and log-rank tests were used for comparison of OS between the two groups (low vs. high expression) for each analyzed protein. Hazard ratios (HR) were estimated based on univariate Cox proportional hazards regression models for each protein and are reported together with corresponding two-sided 95% confidence intervals. Statistical analysis was performed with the Statistical Package for the Social Sciences (SPSS^®^) version 28 (SPSS Inc., IBM Corp.) Statistical significance was considered at a two-sided *p *value of ≤ 0.05.

## Results

Immunohistochemical staining was performed in a cohort of *N* = 65 EC patients. The mean patient’s age of the study cohort was 64.9 (± standard deviation (SD) 11.2) years. 92.3% of the patients displayed endometrioid histology, 4.6% had serous-papillary EC, and 3.1% showed undifferentiated histology. The mean follow-up for the entire cohort was 70.7 months. Data on BMI were available for *N* = 56 patients with a mean BMI of 28.3 (± SD 8.4). A detailed clinicopathologic characterization including histopathologic grading, lymph node involvement and tumor stage according to UICC is presented in Table 1.

Immunohistochemical analyses showed expression of all different m6A writers, readers, and erasers in EC. A strong, predominant nuclear staining for the writers METTL3, METTL14, WTAP, and KIAA1429, the eraser FTO and also the two readers HNRNPC und HNRNPA2B1. YTHDF1-YTHDF3 and METTL4 showed a strong cytoplasmic staining. YTHDC1 displayed a cytoplasmic/membranous and also nuclear staining and ALKBH5 was stained in both, cytoplasm and nucleus. The localization of m6A proteins in different cell compartments reflects the diversity of RNA metabolism.

**Fig. 1:**
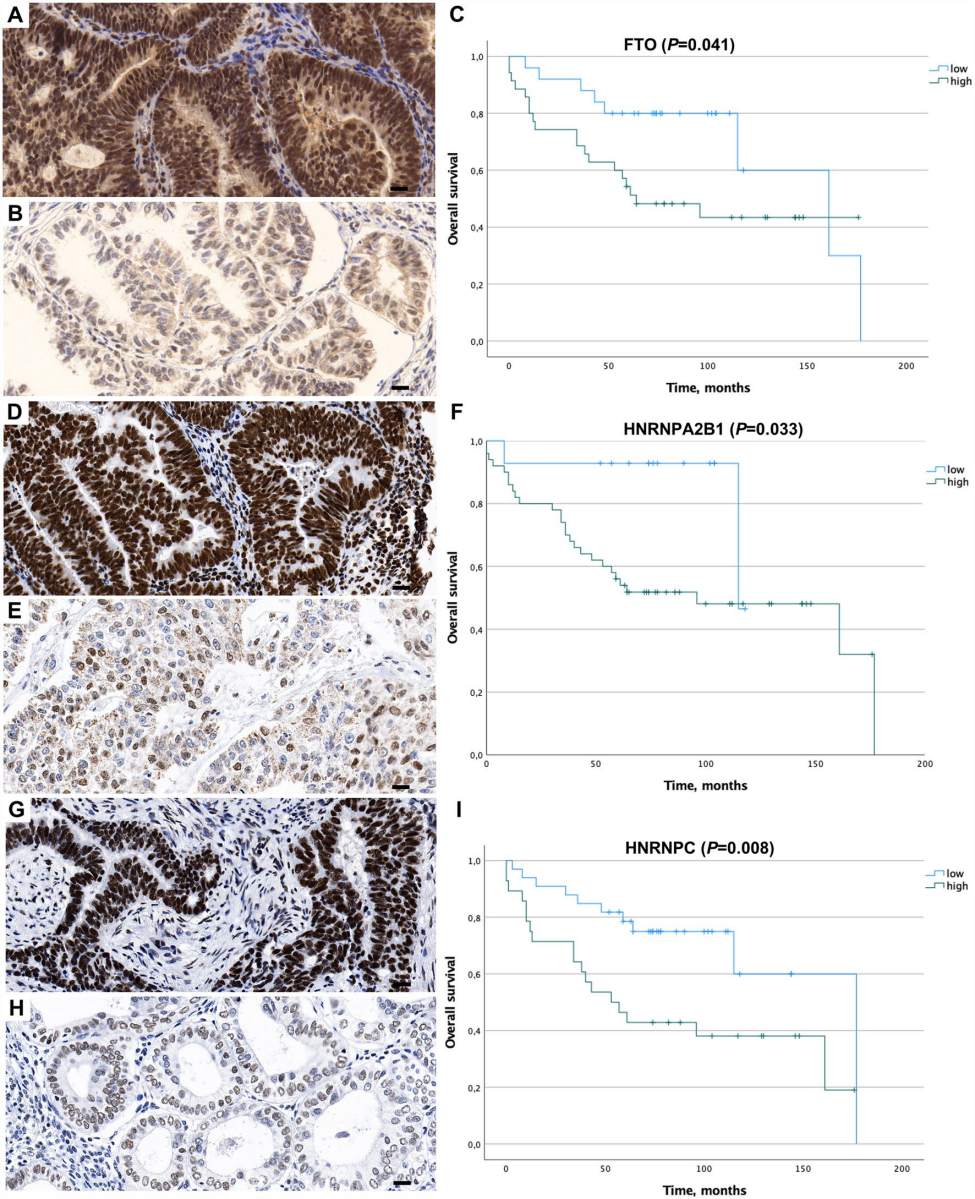
Representative histology sections show high (**A**, **D**, **G**) and low (**B**, **E**, **H**) expression levels of FTO, HNRNPA2B1, and HNRNPC visualized by immunohistochemistry; hematoxylin (blue) was used for nuclear staining (bright field image, 400xmagnification). Kaplan–Meier estimates show a significantly shorter OS (*p* < 0.05) in patients with high nuclear expression of FTO, HNRNPA2B1, and HNRNPC

**Fig. 2:**
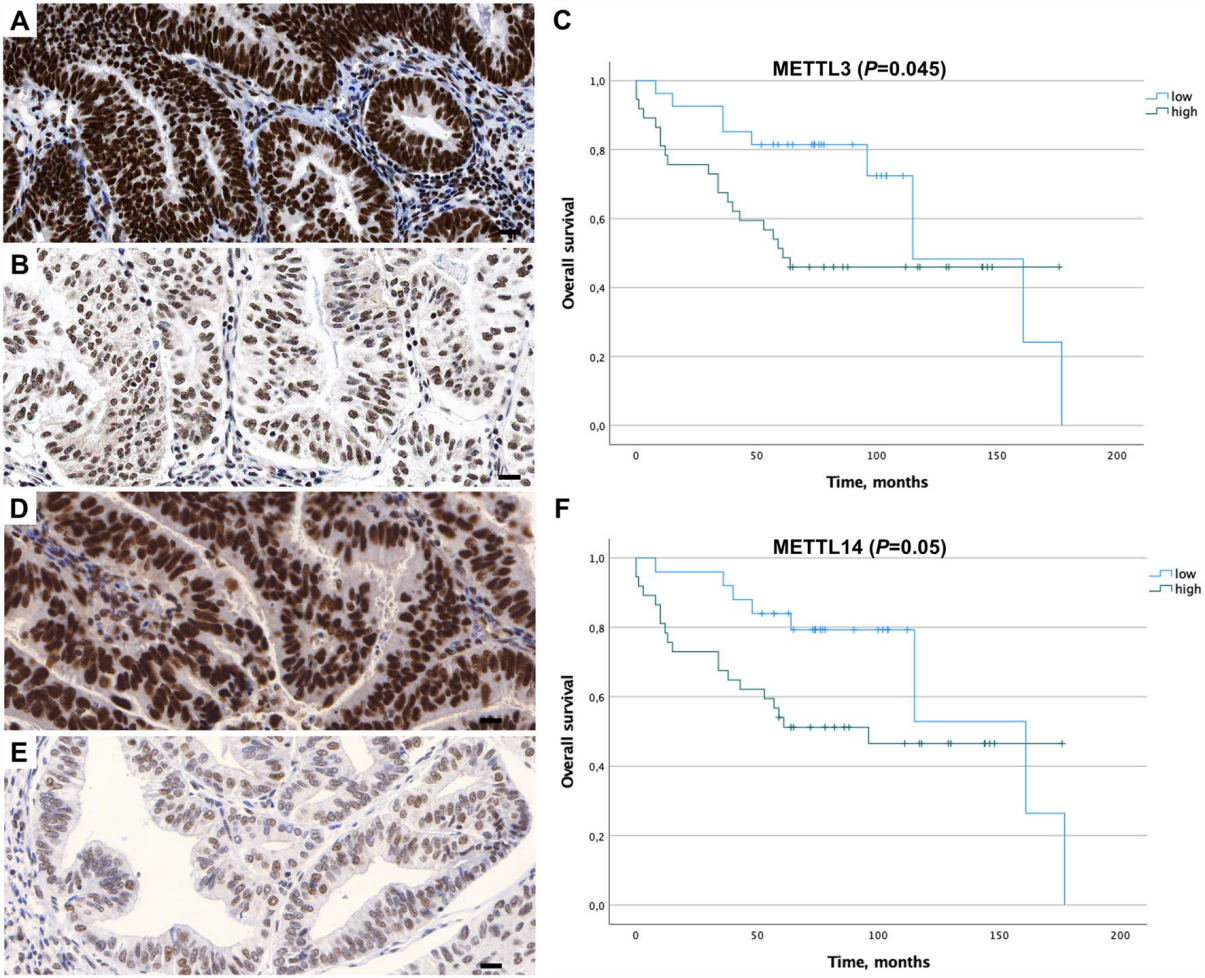
Representative histology sections show high (**A**, **D**) and low (**B**, **E**) expression levels of METTL3 and METTL14 visualized by immunohistochemistry; hematoxylin (blue) was used for nuclear staining (bright field image, 400xmagnification). Kaplan–Meier estimates show a significantly shorter OS (*p* ≤ 0.05) in patients with high nuclear expression of METTL3 and METTL14

In Kaplan–Meier survival analyses, high expression levels of METTL3, METTL14, FTO, HNRNPA2B1, and HNRNPC were significantly associated with a shorter OS (*p* ≤ 0.05; Figs. 1, 2). For all other analyzed proteins, Kaplan–Meier survival analyses indicated a trend toward shorter OS in patients with higher m6A protein expression levels. However, these effects were not statistically significant. A detailed overview of the different m6A proteins with their respective protein expression values is given in Table 2. The prognostic value of METTL3, METTL14, FTO, HNRNPA2B1, and HNRNPC was confirmed in univariate Cox regression analyses. Further correlation analyses of m6A protein expression levels with respect to possible co-factors on survival including grading and lymph node involvement did not show statistically significant values.

In summary, our results point toward dysregulated m6A expression in EC tumorigenesis. In particular, high expression levels of METTL3, MEETTL14, FTO, HNRNPA2B1, and HNRNPC were positively correlated with a shortened OS.

## Discussion

In the present study, we comprehensively evaluated protein expression levels of m6A writers, erasers, and readers in *N* = 65 EC patients. Protein expression data were analyzed with regard to clinical outcomes. We demonstrated that five m6A proteins, namely METTL3, METTL14, FTO, HNRNPA2B1 and HNRNPC, respectively, correlated significantly with a poor OS in EC. In particular, overexpression of the five respective proteins was a negative prognostic marker for survival. This effect was independent of further clinicopathological parameters including histomorphological grading or lymph node involvement.

There is increasing evidence for the crucial impact of mRNA modification in cancer development, metastatic spread, and evolution of drug resistance. The most studied RNA modification is m6A (Ma et al. 2019; Wang et al. 2020). A recent study analyzed mRNA expression data of m6A genes in *N* = 548 EC samples obtained from The Cancer Genome Atlas (TCGA) database. In their analyses, the authors found significant differential expression of all assessed m6A genes in EC tissue compared to normal adjacent tissue (NAT). Moreover, higher m6A mRNA expression levels were detected in poorly differentiated compared to well-differentiated tumors and linked to worse clinical outcomes (Pang et al. 2021). Consistent observations on aberrant m6A in EC were reported by another study based on m6A mRNA expression analysis (Zhang and Yang 2021). In the aforementioned studies and further investigations, it was demonstrated that increased expression of FTO in particular was associated with poorer survival in EC (Zhang et al. 2021; Zhai et al. 2021). In the process of m6A RNA modification, FTO facilitates demethylation of m6A by a complex interplay of oxidizing and converting. Research has shown that FTO promoted metastatic spread in EC via HOXB13 mediated activation of WNT signaling (Zhang et al. 2021). In cervical cancer, FTO enhanced resistance to chemo- and radiotherapy through altered ß-catenin expression caused by m6A demethylation (Zou et al. 2019). This might also be applicable to EC as FTO overexpression might contribute to the failure of radiation and chemotherapy leading to unfavorable clinical outcomes. Of note, FTO expression is closely related to weight gain and obesity (Jia et al. 2008; Smemo et al. 2014). Both are established risk factors for EC development. Thus, FTO overexpression appears to promote EC development both, directly via WNT signaling, and indirectly by increasing of risk factors (Zhang et al. 2021). In our study, we confirmed the prognostic value of FTO expression at the protein level. FTO overexpression was significantly associated with a shortened OS. The data shown render FTO a promising anticancer therapeutic target. Research has shown that inhibition of FTO using MO-I-500, a small molecule inhibitor, effectively suppressed the growth and colony formation of triple negative breast cancer cells (Singh et al. 2016). Recently, data on a more potent FTO inhibitor, namely FB23-2 were published. In the respective study, FB23-2 significantly inhibited AML progression in xenograft transplanted mice (Huang et al. 2019). Interestingly, there are data showing that FTO inhibitors display also anti-obesity effects in vivo and in vitro. The connection between obesity and cancer pathways via FTO seems to be regulated by mammalian target protein rapamycin (mTOR) (Laplante and Sabatini 2012). Entacapone and Epigallocatechin gallate (EGCG) showed in animal disease models additional to the anti-obesity effect a synergistically inhibition of cancer cell lines (Forester and Lambert 2014). However, the aforementioned inhibitors are still in an early preclinical phase but may improve EC therapy in future.

ALKBH5 is the second demethylase involved in m6A modification. ALKBH5 upregulation was found to promote proliferation and invasion of EC cells by activating the IGF1R signaling pathway (Pu et al. 2020). In our analyses, enhanced ALKBH5 expression showed a trend toward a shorter OS but without reaching statistical significance. METTL3 and METTL14 are both responsible in installation of m6A. Whereas METTL14 mainly contributes to the stability of the methylation process, METTL3 is the most important component in catalyzing the transfer of methyl groups to adenine bases in RNA. For both writers, we obtained prognostic values regarding OS in our EC cohort. In pancreatic cancer, absence of METTL3 resulted in increased sensitivity to anticancer treatment, in particular to chemotherapy with gemcitabine, 5-fluoruracil, and platinum (Taketo et al. 2018). Hence, METTL3 overexpression might lead to decreased susceptibility for platinum-based chemotherapy which is applied as first line treatment in advanced EC. In hepatocellular carcinoma (HCC), METTL14 was identified to be involved in the malignant progression of HCC by regulating m6A downstream targets, including cysteine sulfinic acid decarboxylase (CSAD), glutamic- oxaloacetic transaminase 2 (GOT2), and suppressor of cytokine signaling 2 (SOCS2) (Li et al. 2020). Within our analyses, the ‘reader’ HNRNPC, was identified among the m6A enzymes significantly associated with poor survival. In the oncological context, sparse is known regarding the role of HNRNPC. However, elevated expression levels have been observed in HCC, glioblastoma, melanoma, and lung cancer (Wu et al. 2018). Recently, Wu et al. demonstrated suppression of tumor growth by knockdown of HNRNPC in breast cancer cells and a breast cancer xenograft model. These findings suggest a potential oncogene addiction regarding HNRNPC (Wu et al. 2018).

Overall, our study provides further evidence for involvement of m6A RNA modification in EC carcinogenesis. Over-expressions of FTO, METTL3, METTL14, HNRNPA2B1, and HNRNPC in EC are associated with a poor clinical outcome. Molecular biomarkers are gaining increasing importance in the era of individualized oncological therapy. In endometrial cancer, biomarker outperforms classical histomorphological prognostic factors, such as grading and histological subtype, and have entered clinical routine. Biomarkers allow therapy adaptation according to the risk stratification (therapy escalation and therapy de-escalation) (Njoku et al. 2022). To what extent dysregulated m6A RNA modification can be included in the treatment algorithm in EC has to be evaluated in prospective studies. However, our data provide a rationale for integrating m6A into EC biomarker studies. Further, with regard to therapeutic implications, FTO appears to be an interesting target due to the connection of obesity and EC. However, further studies need to be performed to investigate in detail the biological functions and corresponding molecular mechanisms of m6A modifications in EC.

## Conclusion

High expression levels of proteins involved in m6A modification are associated with a poor OS in patients with EC independent of clinicopathological risk parameters. Hence, our study provides further evidence for m6A to serve as a prognostic biomarker and promising target for new anticancer therapeutics in EC.

## Data Availability

The datasets analyzed during the current study are available from the corresponding author on reasonable request.
